# Vaccine-Preventable Infections Among Solid Organ Transplant Recipients in Switzerland

**DOI:** 10.1001/jamanetworkopen.2023.10687

**Published:** 2023-04-28

**Authors:** Laura N. Walti, Catrina Mugglin, Matteo Mombelli, Oriol Manuel, Hans H. Hirsch, Nina Khanna, Nicolas J. Mueller, Christoph Berger, Katia Boggian, Christian Garzoni, Dionysios Neofytos, Christian van Delden, Mirjam Mäusezahl, Cédric Hirzel

**Affiliations:** 1 Department of Infectious Diseases, Bern University Hospital, University of Bern, Bern, Switzerland; 2 Division of Infectious Diseases, Multi-Organ Transplant Program, University Health Network, University of Toronto, Toronto, Ontario, Canada; 3 Transplantation Center and Service of Infectious Diseases, Lausanne University Hospital, Lausanne, Switzerland; 4 Division of Infectious Diseases and Hospital Epidemiology, University Hospital Basel, Basel, Switzerland; 5 Division of Infectious Diseases and Hospital Epidemiology, University Hospital Zurich and University Zurich, Zurich, Switzerland; 6 Division of Infectious Diseases and Hospital Epidemiology, University Children’s Hospital Zurich, Zurich, Switzerland; 7 Division of Infectious Diseases and Hospital Hygiene, Cantonal Hospital St Gallen, St Gallen, Switzerland; 8 Clinic of Internal Medicine and Infectious Diseases, Clinica Luganese, Lugano, Switzerland; 9 Transplant Infectious Diseases Unit, University Hospitals of Geneva and Faculty of Medicine, Geneva, Switzerland; 10 Swiss Federal Office of Public Health, Epidemiological Evaluation and Surveillance Section, Bern, Switzerland

## Abstract

**Importance:**

Vaccine responses are decreased in solid organ transplant (SOT) recipients, and given the complexity of implementation, vaccination programs may be suboptimal. The actual burden of vaccine-preventable infections (VPIs) among SOT recipients remains unclear.

**Objectives:**

To assess the incidence rate of VPIs among SOT recipients and to evaluate whether SOT recipients are at increased risk for specific VPIs compared with the general population.

**Design, Setting, and Participants:**

This nationwide cohort study used data from the Swiss Transplant Cohort Study on VPIs in individuals who underwent SOT from May 2008 to June 2019 (follow-up until December 2019) and data from the Swiss Federal Office of Public Health on notifiable VPIs in the general population in the same period. Data were analyzed from January 2021 to June 2022.

**Exposures:**

Solid organ transplant.

**Main Outcomes and Measures:**

The main outcomes were the incidence rate of the following VPIs in SOT recipients: hepatitis A and B, diphtheria, *Haemophilus influenzae* infection, influenza, measles, mumps, pertussis, pneumococcal disease, poliomyelitis, meningococcal disease, rubella, tetanus, tick-borne encephalitis, and varicella zoster virus infection. Age-adjusted standardized incidence ratios were used to assess whether VPIs occurred more frequently in SOT recipients compared with the general population. For SOT recipients, factors associated with occurrence of VPIs were explored and the associated morbidity and mortality assessed.

**Results:**

Of 4967 SOT recipients enrolled (median age, 54 years [IQR, 42-62 years]; 3191 [64.2%] male), 593 (11.9%) experienced at least 1 VPI. The overall VPI incidence rate was higher in the population that underwent SOT (30.57 per 1000 person-years [PY]; 95% CI, 28.24-33.10 per 1000 PY) compared with the general population (0.71 per 1000 PY). The standardized age-adjusted incidence ratio for notifiable VPIs in SOT recipients was higher compared with the general population (27.84; 95% CI, 25.00-31.00). In SOT recipients, influenza and varicella zoster virus infection accounted for most VPI episodes (16.55 per 1000 PY [95% CI, 14.85-18.46 per 1000 PY] and 12.83 per 1000 PY [95% CI, 11.40-14.44 per 1000 PY], respectively). A total of 198 of 575 VPI episodes in the population that underwent SOT (34.4%) led to hospital admission, and the occurrence of a VPI was associated with an increased risk for death and/or graft loss (hazard ratio, 2.44; 95% CI, 1.50-3.99; *P* = .002). In multivariable analysis, age 65 years or older at the time of transplant (incidence rate ratio [IRR], 1.29; 95% CI, 1.02-1.62) and receipt of a lung (IRR, 1.77; 95% CI, 1.38-2.26) or a heart (IRR, 1.40; 95% CI, 1.05-1.88) transplant were associated with an increased risk of VPI occurrence.

**Conclusions and Relevance:**

In this study, 11.9% of SOT recipients experienced VPIs, and the incidence rate was higher than in the general population. There was significant morbidity and mortality associated with these infections in the population that underwent SOT, which highlights the need for optimizing immunization strategies.

## Key Points

**Question:**

What is the incidence rate of vaccine-preventable infections in solid organ transplant (SOT) recipients compared with the general population?

**Findings:**

In this nationwide cohort study of 4967 SOT recipients in Switzerland, the incidence rate of vaccine-preventable infections was higher among SOT recipients compared with the general population.

**Meaning:**

The findings suggest that there is an important need for optimization of vaccine strategies in SOT recipients.

## Teaser

This cohort study assesses the incidence rate of vaccine-preventable infections overall and by pathogen in solid organ transplant recipients compared with the general population in Switzerland.

## Introduction

Infectious diseases are important causes of morbidity and mortality in immunocompromised patients, such as solid organ transplant (SOT) recipients.^1^ Vaccination has proven to be an efficient way of preventing communicable diseases for over a century. However, decreased immunogenicity of vaccines in the posttransplant setting,^2^ underimmunization due to logistic difficulties before transplant,^3,4,5,6^ vaccine hesitancy,^7^ and imperfect financial coverage^8^ may be associated with suboptimal vaccine coverage and reduced vaccine effectiveness in transplant recipients. Therefore, SOT recipients might be at increased risk for vaccine-preventable infections (VPIs).

In a study of pediatric SOT recipients in the US,^9^ 15% of patients had a VPI within the first 5 years after transplant. The costs of initial transplant hospitalizations complicated by VPIs were higher compared with those not complicated by VPIs. Similar findings were reported for heart transplant recipients.^10^ However, whether SOT recipients are at increased risk for infections with vaccine-preventable pathogens in comparison with the general population remains largely unexplored.^11^ Influenza,^12,13,14^ varicella zoster virus (VZV) infection,^15,16^ and invasive pneumococcal disease (IPD)^17,18,19^ have been identified as important VPIs in the posttransplant setting, and having a lung transplant was previously shown to be associated with an increased risk for influenza compared with other transplanted organs.^14^

Epidemiological studies including both the general population and SOT recipients might be useful to identify specific VPIs that are more likely to occur in the population undergoing transplant and may provide important information for future interventional studies to evaluate targeted immunization strategies for SOT recipients. We (1) assessed the incidence rate of VPIs in a nationwide cohort of SOT recipients in Switzerland, (2) compared the incidence rate of notifiable VPIs (NVPIs) between SOT recipients and the entire population of Switzerland, and (3) assessed potential factors, morbidity, and mortality associated with VPIs in the population undergoing SOT. All analyses were prespecified, and we hypothesized that VPI incidence would be higher in SOT recipients compared with the general population.

## Methods

### Swiss Transplant Cohort Study

We conducted a cohort study based on data from the nationwide Swiss Transplant Cohort Study (STCS).^20^ All 6 Swiss transplant centers participate in the STCS, and for the analyzed period, around 93% of transplant recipients in Switzerland were included. A detailed cohort description of the STCS was published previously.^20,21^ Infections were classified according to standardized definitions as previously described.^1^ Each infection episode was validated by a transplant infectious diseases specialist. Data on demographic characteristics, type of transplant, immunosuppressive regimen, occurrence of VPI, rejection episodes and treatments, and outcomes of VPIs (hospitalization [only documented for episodes after 2012], graft loss, and death) were extracted from the STCS database (eTable 1 in Supplement 1). In the STCS, ethnicity information is self-reported by the study participants; we included information about ethnicity to help address potential limitations in generalizability. The STCS and the current substudy were approved by each local ethics committee of participating centers (Ethics Commission of the Canton of Bern, Bern, Switzerland). All study participants of the STCS provided written informed consent. This study followed the Strengthening the Reporting of Observational Studies in Epidemiology (STROBE) reporting guideline.^22^

### Study Participants

We included all recipients of lung, heart, liver, kidney, and kidney-pancreas grafts who underwent transplant from May 2008 to June 2019. The recipients were followed up until December 2019 to allow for a minimal follow-up time of 6 months after transplant. Data were extracted in December 2020. Only data related to the first transplant were analyzed. Anonymized data on NVPIs in the general population were provided by the Swiss Federal Office of Public Health (data extracted in February 2021). For data protection reasons, characteristics other than age and the specific VPI were not available for the general population.

### Outcome Variable

The primary outcome was the incidence rate of VPIs among SOT recipients. We considered all of the following infections as VPIs: hepatitis A and B, diphtheria, *Haemophilus influenzae* infection, influenza, measles, mumps, pertussis, pneumococcal disease, poliomyelitis, meningococcal disease, rubella, tetanus, tick-borne encephalitis (TBE), and VZV infection. Although current vaccines do not cover all serotypes of *Streptococcus pneumoniae* and *H influenzae* and *H influenzae* type b booster vaccination is not recommended for adult SOT recipients in Switzerland, we considered these infections as VPIs. Human papilloma virus (HPV) infection was not included since precancerous HPV-associated lesions are not systematically recorded in the STCS.

We also compared the incidence rate of NVPIs in SOT recipients and the general population. In Switzerland, the following VPIs are mandatory to report to health authorities: hepatitis A and B, diphtheria, invasive *H influenzae* infection, laboratory-confirmed influenza, measles, IPD, poliomyelitis, invasive meningococcal disease, tetanus, and TBE (eTable 2 in Supplement 1). We did not compare primary hepatitis B infection incidence rates in SOT recipients and the general population due to differences in reporting; the definition of the STCS requires documentation of seroconversion. This information is not available for the general population. Secondary outcomes included factors, morbidity (hospital admission, graft loss), and mortality associated with VPIs in the cohort of SOT recipients.

### Statistical Analysis

Data were analyzed from January 2021 to June 2022. For the SOT population, the VPI incidence rates are presented as point estimates and corresponding 95% CIs (estimated using negative binomial regression). For the incidence rates of NVPIs in the general population, a complete data set of the entire Swiss population was available. These incidence rates are not estimates, and therefore, no 95% CIs are presented. As VPI incidence rates varied with age,^23,24^ we calculated age-adjusted standardized incidence ratios (SIRs) and corresponding 95% CIs to compare the incidence rate of NVPI in the general population with that among SOT recipients.^25^

To account for the fact that patients may experience multiple episodes of VPIs, we used mixed-effects negative binomial regression to calculate incidence rates and to investigate the association of baseline patient characteristics (age, sex, transplanted organ, and transplant induction treatment) with VPI occurrence.^26^ We included all VPI episodes, accounted for recurring events using individual-level clustering, and included time of exposure for each individual in the model.

To assess whether treatment of a rejection episode was associated with an increased risk for VPIs in the following 3 months, we used a time-dependent Cox proportional hazards regression model.^27^ We adjusted the analysis for the type of transplant, induction therapy, sex, and age. Rejection treatment was considered a time-varying covariate. We used a time-dependent Cox proportional hazards regression model (adjusted for the type of transplant, sex, and age, with VPI as time-varying covariate) to assess the association of VPI occurrence with graft loss or death in the following 3 months. Proportional hazards assumptions were verified by plotting Schoenfeld residuals. None of the variables used included missing values.

Statistical analysis was conducted using Stata, version 15 (StataCorp LLC) and R, version 4.0.3 (R Project for Statistical Computing). Figures were plotted in R, version 4.0.3 and in GraphPad Prism, version 8.0. Two-sided *P* < .05 was considered significant.

## Results

A total of 4967 SOT recipients (2784 [56.0%] kidney, 1100 [22.1%] liver, 454 [9.1%] lung, 385 [7.8%] heart, and 244 [4.9%] combined) were included. Median age was 54 years (IQR, 42-62 years); 3191 (64.2%) were male, and 1776 (35.8%) were female. A total of 151 SOT recipients (3.0%) were African; 184 (3.7%), Asian; 4551 (91.6%), White; and 64 (1.3%), other (1 [<0.1%] Malagasy, 3 [<0.1%] Mauritian descent, 22 [0.4%] Middle Eastern, 5 [0.1%] multiethnic, 5 [0.1%] North African descent, and 28 [0.6%] South American or Caribbean); 17 (0.3%) had unknown ethnicity. Patient characteristics of SOT recipients with and without VPIs are detailed in Table 1.

**Table 1.** Characteristics of 4967 Patients Who Underwent SOT

Characteristic	SOT recipients^a^	
	Without VPI (n = 4374)	With VPI (n = 593)
Age at transplant, y		
Median (IQR), y	54 (42-62)	53 (41-62)
<18	232 (5.3)	40 (6.7)
18-64	3542 (81.0)	474 (79.9)
≥65	600 (13.7)	79 (13.3)
Sex		
Female	1539 (35.2)	237 (40.0)
Male	2835 (64.8)	356 (60.0)
Ethnicity		
African	134 (3.1)	17 (2.9)
Asian	159 (3.6)	25 (4.2)
White	4005 (91.6)	546 (92.1)
Other^b^	59 (1.4)	5 (0.8)
Unknown	17 (0.4)	0
Follow up, median (IQR), y	3.85 (1.15-6.90)	6.06 (3.82-8.51)
Organ transplanted		
Kidney	2425 (55.4)	359 (60.5)
Liver	1025 (23.4)	75 (12.6)
Heart	322 (7.4)	63 (10.6)
Lung	378 (8.6)	76 (12.8)
Combined	224 (5.1)	20 (3.4)
Induction regiment contained		
Basiliximab or other	3279 (75.0)	417 (70.3)
ATG	925 (21.1)	146 (24.6)
Rituximab	166 (3.8)	30 (5.1)
Maintenance immunosuppression		
Glucocorticosteroids	4214 (96.3)	563 (94.9)
MMF	3744 (85.6)	535 (90.2)
Azathioprine	120 (2.7)	15 (2.5)
Cyclosporine	922 (21.1)	151 (25.5)
Tacrolimus	3138 (71.7)	390 (65.8)
Everolimus	45 (1.0)	10 (1.7)
Sirolimus	20 (0.5)	0
Treated rejection episode		
Yes	1096 (25.1)	211 (35.6)
No	3278 (74.9)	382 (64.4)
Treated rejection episodes, median (IQR), No.	0 (0-1)	0 (0-1)

Abbreviations: ATG, antithymocyte globulins; MMF, mycophenolate mofetil; SOT, solid organ transplant; VPI, vaccine-preventable infection.

^a^ Data are presented as number (percentage) of recipients unless otherwise indicated. Due to rounding, percentages in some columns may not add to 100%.

^b^ Other includes Malagasy (n = 1), Mauritian descent (n = 3), Middle Eastern (n = 22), multiethnic (n = 5), North African descent (n = 5), and South American or Caribbean (n = 28).

### VPIs in SOT Recipients

**Figure 1.**
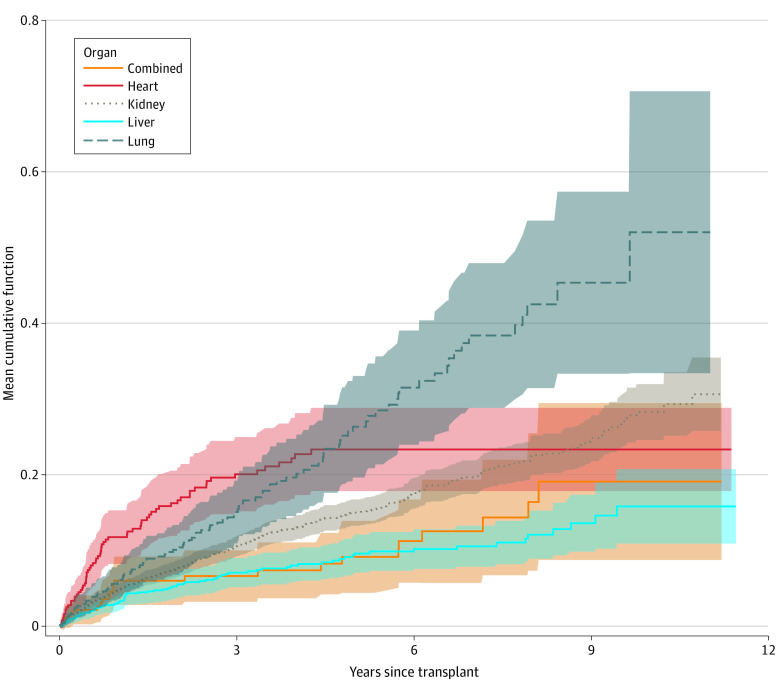
Mean Cumulative Vaccine-Preventable Infection Episodes per Solid Organ Transplant Recipient Stratified by Type of Transplanted Organs Mean cumulative function is equivalent to the mean cumulative number of vaccine-preventable infections per solid organ transplant recipient. Shading indicates 95% CIs.

We identified 668 VPI episodes in 593 SOT recipients (11.9%) (eFigure in Supplement 1). Most VPIs occurred late (>1 year) after transplant (eTable 3 in Supplement 1). Influenza (360 episodes [53.9%] in 333 patients [6.7%]), VZV infection (282 episodes [42.2%] in 269 patients [5.4%]), and IPD (10 episodes [1.5%] in 9 patients [0.2%]) were the most common VPIs (Table 2). Noninvasive pneumococcal infections (infections lacking isolation of *S pneumoniae* from sterile sites, eg, pneumonia with a positive urinary antigen test result or positive sputum culture result) outnumbered invasive cases (57 of 4967 [1.1%]). This was similar for *H influenzae* infection (invasive cases: 6 of 4967 [0.1%]; noninvasive cases: 90 of 4967 [1.8%]). No cases of hepatitis A, measles, mumps, poliomyelitis, rubella, diphtheria, or tetanus were identified in SOT recipients. Recipients of a lung or heart transplant had a higher VPI incidence rate compared with kidney or liver transplant recipients (Figure 1). Similarly, influenza incidence rate was highest in lung transplant recipients (40.46 [95% CI, 31.97-51.70] per 1000 person-years [PY]) and lowest in liver transplant recipients (8.82 [95% CI, 6.13-12.67] per 1000 PY).

**Table 2.** Vaccine-Preventable Infection–Associated Morbidity and Mortality in 4967 Solid Organ Transplant Recipients

Disease	Episodes, No.	Patients, No. (%) (N = 4967)	Incidence rate, per 1000 person-years (95% CI)	Episodes, No./total No. (%)			
				Hospitalized for VPI^a^	Graft loss within 90 d after VPI	Death within 30 d after VPI
Overall	668	593 (11.9)	30.57 (28.24-33.10)	198/575 (34.4)	6/668 (0.9)	7/668 (1.0)
Viral VPI						
All	649	578 (11.6)	29.70 (27.41-32.18)	183/558 (32.8)	3/642 (0.5)	7/649 (0.1)
VZV	282	269 (5.4)	12.83 (11.40-14.44)	57/226 (25.2)	3/282 (1.1)	2/282(0.7)
Influenza	360	333 (6.7)	16.55 (14.85-18.46)	124/325 (38.2)	3/360 (0.8)	4/282 (1.4)
HBV infection	5	5 (0.1)	0.23 (0.09-0.54)	0/5	0/5	0/5
TBE	2	2 (<0.1)	0.09 (0.02-0.36)	2/2 (100)	0/2	1/2 (50.0)
Bacterial VPI						
All	19	18 (0.4)	0.87 (0.53-1.39)	15/17 (88.2)	0/19	0/19
IPD	10	9 (0.2)	0.45 (0.23-0.90)	10/10 (100)	0/10	0/10
IHI	6	6 (0.1)	0.27 (0.12-0.61)	4/4 (100)	0/6	0/6
IMD	1	1 (<0.1)	0.04 (0.01-0.32)	1/1 (100)	0/1	0/1
Pertussis	2	2 (<0.1)	0.09 (0.02-0.36)	0/2	0/2	0/2

Abbreviations: HBV, hepatitis B virus; IHI, invasive *Haemophilus influenzae* infection; IMD, invasive meningococcal disease; IPD, invasive pneumococcal disease; TBE, tick-borne encephalitis; VPI, vaccine-preventable infection; VZV, varicella zoster virus.

^a^ Data on hospital admission were only available from December 2011 to December 2019 (575 patients).

Age 65 years or older (incidence rate ratio [IRR], 1.29; 95% CI, 1.02-1.62) and transplant of lung or heart compared with kidney transplant (lung transplant: IRR, 1.77 [95% CI, 1.38-2.26]; heart transplant: IRR, 1.40 [95% CI, 1.05-1.88]) were associated with increased risk of VPI occurrence. Liver transplant was associated with a decreased VPI risk (IRR, 0.62; 95% CI, 0.48-0.80) compared with kidney transplant (Table 3). The type of induction treatment was not associated with VPI occurrence (Table 3).

**Table 3.** Factors Associated With Vaccine-Preventable Infection Occurrence in Solid Organ Transplant Recipients

Variable	IRR (95% CI)	*P* value
Sex		
Female	1.15 (0.98-1.36)	.08
Male	1 [Reference]	NA
Age, y		
<18	1.27 (0.92-1.75)	.16
18-64	1 [Reference]	NA
≥65	1.29 (1.02-1.62)	.03
Organ transplant		
Kidney	1 [Reference]	NA
Liver	0.62 (0.48-0.80)	<.001
Lung	1.77 (1.38-2.26)	<.001
Heart	1.40 (1.05-1.88)	.02
Combined	0.72 (0.45-1.15)	.17
Induction therapy		
Basiliximab or other	1 [Reference]	NA
ATG	1.04 (0.84-1.30)	.70
Rituximab	1.14 (0.79-1.65)	.47

Abbreviations: ATG, antithymocyte globulins; IRR, incidence rate ratio; NA, not applicable.

Rejection treatment was not associated with an increased risk for VPIs in the following 3 months (hazard ratio, 1.26; 95% CI, 0.90-1.76) (eTable 4 in Supplement 1). Compared with kidney transplant recipients, lung transplant recipients were at increased risk for influenza (IRR, 2.51; 95% CI, 1.88-3.34), and heart transplant was associated with an increased risk for VZV (IRR, 1.72; 95% CI, 1.16-2.55) (eTables 5 and 6 in Supplement 1).

### Morbidity and Mortality Associated With VPIs in SOT Recipients

Overall admission rates among SOT recipients due to VPIs were 34.4% (198 of 575 episodes; data available from December 2011 to December 2019). All patients with IPD episodes (10 of 10 patients) and invasive *H influenzae* infection (4 of 4) were admitted. Notably, hospitalization rates were similar for IPD and noninvasive pneumococcal infection episodes (100% [10 of 10] vs 73.7% [42 of 57]; *P* = .10). The same was true for invasive and noninvasive *H influenzae* infection episodes (100% [4 of 4] vs 87.7% [50 of 57]; *P* > .99). Influenza (38.2% [124 of 325 patients]) and VZV infection (25.2% [57 of 226]) also frequently led to hospital admission. The 30-day mortality rate after a VPI was 1.1% (7 of 668 episodes); 4 patients died in the first month following influenza, 2 following VZV, and 1 following TBE (Table 2). The graft loss rate was 0.9% (6 of 668 episodes) in the 3 months following a VPI. Occurrence of a VPI was associated with an increased risk for graft loss and/or death in the following 30 days (hazard ratio, 2.44; 95% CI, 1.50-3.99) (eTable 7 in Supplement 1).

### VPI Incidence Rates in SOT Recipients Compared With the General Population

**Figure 2.**
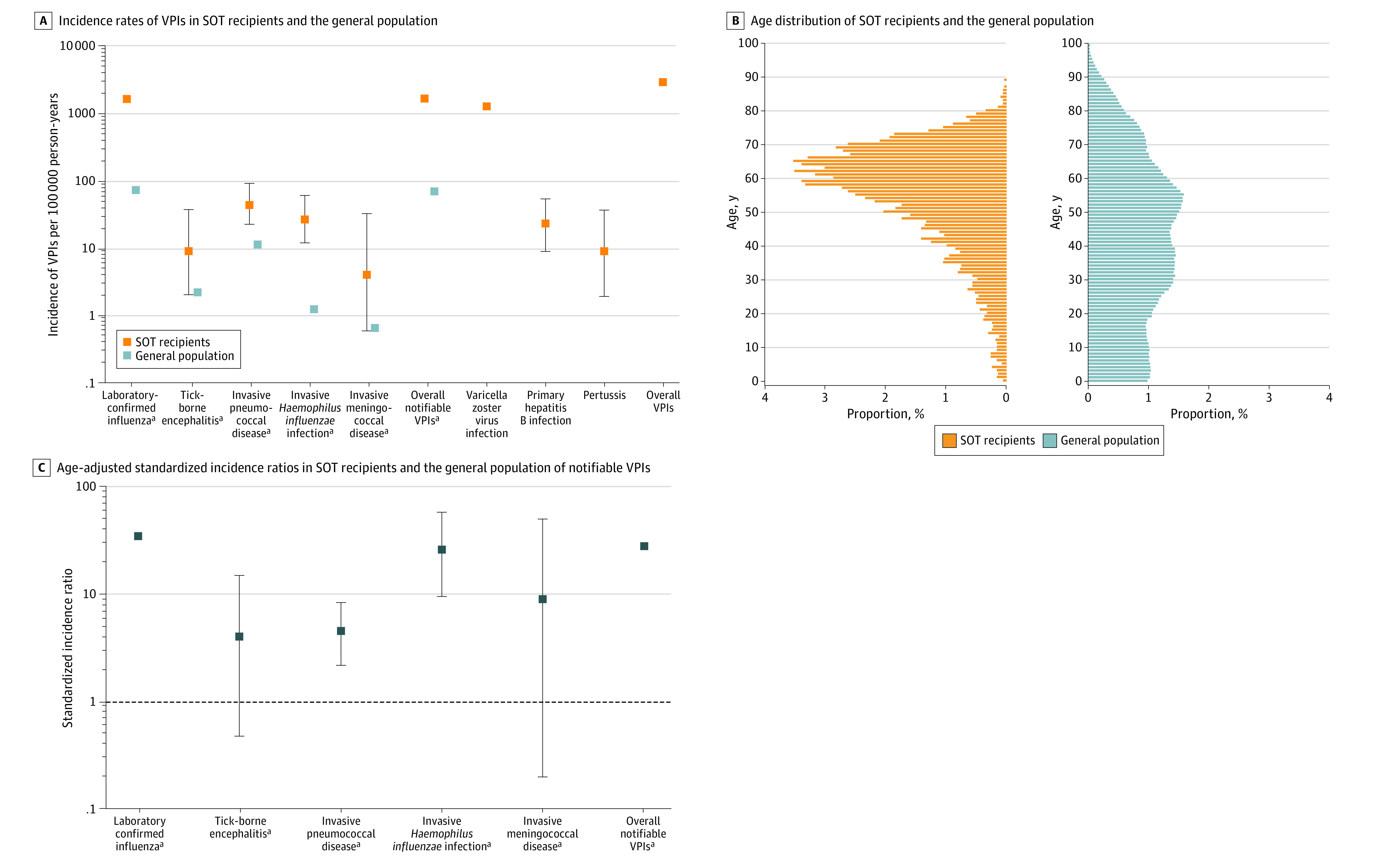
Vaccine-Preventable Infections (VPIs) in Solid Organ Transplant (SOT) Recipients and the General Population A, Due to the logarithmic scale of the x-axis, 95% CIs for SOT recipients are too narrow to be visible for infections with an incidence rate greater than 1000 per 100 000 person-years. A and C, Whiskers represent 95% CIs. ^a^Data for SOT recipients and the general population.

The VPI with the highest incidence rate in SOT recipients was influenza (16.55 per 1000 PY; 95% CI, 14.85-18.46 per 1000 PY) followed by VZV (12.83 per 1000 PY; 95% CI, 11.40-14.44 per 1000 PY) and IPD (0.45 per 1000 PY; 95% CI, 0.23-0.90 per 1000 PY) (Figure 2A and Table 2). For the general population, only data on NVPIs were available. The overall NVPI incidence rate was higher in the population that underwent SOT (30.57 per 1000 PY; 95% CI, 28.24-33.10 per 1000 PY) compared with the general population (0.71 per 1000 PY). Among these diseases, influenza infection was the most common (0.56 per 1000 PY), followed by IPD (0.11 per 1000 PY) and TBE (0.02 per 1000 PY) (Figure 2A). Due to substantial differences in the age distribution of SOT recipients and the general population (Figure 2B), we used age-adjusted SIRs to compare incidence rates of NVPIs between the 2 populations. NVPIs occurred more frequently in SOT recipients compared with the general population (SIR, 27.84; 95% CI, 25.00-31.00) (Figure 2C). Age-adjusted incidence rates for laboratory-confirmed influenza (SIR, 34.1; 95% CI, 31.00-38.00), IPD (SIR, 4.54; 95% CI, 2.18-8.35), and invasive *H influenzae* infection (SIR, 26.1; 95% CI, 9.50-57.00) were significantly higher in the SOT population. Incidence rates for invasive meningococcal disease (SIR, 9.1; 95% CI, 0.20-50.00) and TBE (SIR, 4.03; 95% CI, 0.48-15.00) were not significantly different between the 2 populations.

## Discussion

The major findings of our study were as follows. First, more than 10% of SOT recipients experienced at least 1 VPI episode. Second, influenza and VZV were the most frequent VPIs affecting SOT recipients and were associated with significant health care use. Third, the occurrence of a VPI was associated with an increased risk for graft loss or death in SOT recipients. Fourth, the overall rate of notifiable VPIs was more than 27 times higher in SOT recipients compared with the general population, mostly driven by higher incidence rates of influenza, IPD, and invasive *H influenzae* infection among SOT recipients. Fifth, recipients of lung and heart transplants and patients 65 years or older were most likely to experience VPIs.

Despite current efforts including systematic immunization evaluations before transplant at all study centers and the availability of immunization guidelines for SOT recipients,^28,29^ 11.9% of SOT recipients in Switzerland experienced at least 1 VPI episode. Infection with a vaccine-preventable pathogen was associated with health care utilization. A previous pediatric study showed that hospitalization rates due to VPIs were higher in transplant recipients compared with control individuals who did not undergo transplant^9^; similar findings were published for heart transplant recipients.^10^ In our analysis, VPIs occurred more frequently among SOT recipients than in the general population. This finding highlights the importance of ongoing efforts to decrease the incidence of these potentially preventable infections, including attempts to increase vaccine uptake in transplant recipients^5,30,31^ and to improve the immunogenicity of vaccines in immunocompromised patients.^32,33,34,35,36^

Laboratory-confirmed influenza affected 6.7% of transplant recipients in our study. The influenza incidence rate was highest in lung transplant recipients (40.51 per 1000 PY) and lowest in liver transplant recipients (8.47 per 1000 PY). These findings are comparable with previous data.^11,14^ The influenza-associated hospitalization rate was 38% in our study, similar to the rate in a recent cohort study from Australia^11^ but lower than previously reported in a multicenter study^12^ in which two-thirds of SOT recipients were admitted to the hospital. The lower admission rate in our study compared with the study by Kumar et al^12^ may be explained by the lower proportion of lung transplant recipients in the present analysis, and differences in health care systems may have also contributed to disparities in hospitalization rates. The age-adjusted SIR for influenza was higher in transplant recipients than in the general population. This finding is in line with the results of an Australian study that reported SIRs of 7 to 40 (depending on the influenza season, with a decreasing trend over time).^11^ Our data suggest that the burden of influenza in transplant recipients is substantial and that efforts toward improving influenza prevention seem to be urgently needed.

Varicella zoster infection affected 5.4% of patients in the present cohort. This is slightly lower compared with historic studies.^16,37^ With the recent introduction of an adjuvanted recombinant subunit herpes zoster vaccine that proved to be immunogenic in the posttransplant setting,^38,39^ the VZV infection rate may further decline in the near future.

Compatible with the findings of other studies,^11,19^ we found that IPD was relatively uncommon in the population that underwent SOT. The incidence rate of IPD in SOT recipients and in the general population was lower compared with that in a study that assessed IPD in the late 1990s and early 2000s.^18^ Over the past 2 decades, a steady decrease in the rate of IPD has been observed in the general population.^40^ This decrease may be explained by the introduction of effective (pediatric) pneumococcal vaccines.^40,41^

Invasive *H influenzae* infection was rare in the present cohort. The only other study, to our knowledge, that assessed the *H influenzae* infection incidence rate in SOT recipients reported similar findings.^19^ Similar to pneumococcal infections, noninvasive infections outnumbered invasive *H influenzae* infections. Specific strategies may include pre- and posttransplant boosters, which are not recommended by national^29^ or international guidelines.^28^

The incidence rates of meningococcal disease and TBE were not different between SOT recipients and the general population, although it has to be mentioned that the sample size of the cohort of SOT recipients might have been inappropriate to detect differences for rare infections. Our findings concerning invasive meningococcal disease support the recommendations of the current immunization guidelines for SOT recipients, which do not routinely recommend meningococcal immunization or reimmunization before or after transplant.^28,29^

In our study, age 65 years or older and receipt of a lung transplant were associated with a higher VPI incidence rate. This might be due to impaired immune responses in older individuals^42,43^ and the usually higher target levels of immunosuppression in lung transplant recipients.

### Strengths and Limitations

The main strengths were (1) the high proportion of SOT recipients included (>90% of all Swiss SOT recipients), (2) the prospective data collection of inpatient and outpatient data, and (3) the harmonized identification procedures for infections. A limitation of our study is that data on immunization were not systematically collected by the STCS. We can therefore not provide information on pre- and posttransplant vaccination of SOT patients. There was no uniform approach for vaccination of SOT recipients before the availability of Swiss national guidelines in 2014.^29^ This might have also contributed to the increased SIR of NVPIs in the population that underwent SOT. We are confident that the STCS study population is representative of the entire community of SOT recipients in Switzerland. However, we cannot definitively exclude systematic differences between the small fraction of SOT recipients (approximately 7%) who were not enrolled in the STCS and the STCS study population. In addition to information for age, detailed demographic data were not available for individuals in the general population. This precluded adjustment in the analysis for VPI IRRs (SOT recipients vs general population) for other factors possibly associated with VPI occurrence. In addition, the mode of data collection for VPIs differed between SOT recipients and the general population.^1,21,44^ However, the definitions used for the respective VPIs in the cohorts were stringent; both were based on laboratory confirmation. The threshold for influenza virus testing might be lower for SOT recipients. As we relied on laboratory-confirmed influenza infection, it is likely that the influenza incidence rate in the general population was underestimated. In addition, we did not restrict invasive pneumococcal, meningococcal, and *H influenzae* infections to vaccine-preventable serotypes. In Switzerland, serotyping is performed at the national reference laboratory, and we did not have access to these data. Furthermore, *H influenzae* type b vaccination is not recommended for adult SOT recipients in Switzerland. Despite this, we considered the infection to be vaccine preventable. Moreover, the STCS does not systematically collect data on precancerous HPV-associated lesions. We therefore could not provide information on this potentially underrecognized VPI in the population of SOT recipients. For the general population, only data on NVPIs were available. Consequently, we had to restrict the comparison of incidence rates of VPIs between SOT recipients and the general population to these notifiable infections.

## Conclusions

This study found that VPIs were common after SOT. Despite current efforts, 11.9% of recipients experienced VPIs. The overall incidence rate of NVPIs in the SOT population was higher than that in the general population, including the incidence rates for influenza, IPD, and *H influenzae* infection. These findings suggest that efforts for optimization of vaccine strategies in SOT recipients should focus on VPIs with either a high incidence in this particular population or a higher incidence rate compared with the general population.

## Notes

Supplement 1. **eTable 1.** Data Sources (STCS/FOPH) **eTable 2.** VPI Definitions (STCS/FOPH) **eTable 3.** Time From Transplant to VPI Occurrence **eTable 4.** Risk Factors for VPI Occurrence in Solid Organ Transplant Recipients **eTable 5.** Risk Factors for Influenza Occurrence in Solid Organ Transplant Recipients **eTable 6.** Risk Factors for VZV Occurrence in Solid Organ Transplant Recipients **eTable 7.** Association of VPI Occurrence and Graft-Loss/Death During the First 90 Days Post-Transplant **eFigure.** Vaccine Preventable Infections in 4967 Solid Organ Transplant Recipients

Supplement 2.
Swiss Transplant Cohort Study

Supplement 3.
Data Sharing Statement
