# Supplementary material for: Vaccine-Preventable Infections Among Solid Organ Transplant Recipients in Switzerland
Source: JAMA Netw Open. 2023 Apr 28;6(4):e2310687. doi: 10.1001/jamanetworkopen.2023.10687 (PMC10148200; doi:10.1001/jamanetworkopen.2023.10687)
Supplement: Supplement 1. — eTable 1. Data Sources (STCS/FOPH) eTable 2. VPI Definitions (STCS/FOPH) eTable 3. Time From Transplant to VPI Occurrence eTable 4. Risk Factors for VPI Occurrence in Solid Organ Transplant Recipients eTable 5. Risk Factors for Influenza Occurrence in Solid Organ Transplant Recipients eTable 6. Risk Factors for VZV Occurrence in Solid Organ Transplant Recipients eTable 7. Association of VPI Occurrence and Graft-Loss/Death During the First 90 Days Post-Transplant eFigure. Vaccine Preventable Infections in 4967 Solid Organ Transplant Recipients [file jamanetwopen-e2310687-s001.pdf]

Supplemental Online Content

Walti LN, Mugglin C, Mombelli M, et al; Swiss Transplant Cohort Study. Vaccine-preventable infections among solid organ transplant recipients in Switzerland. *JAMA Netw Open.* 2023;6(4):e2310687. doi:10.1001/jamanetworkopen.2023.10687

eTable 1. Data Sources (STCS/FOPH)

eTable 2. VPI Definitions (STCS/FOPH)

eTable 3. Time From Transplant to VPI Occurrence

eTable 4. Risk Factors for VPI Occurrence in Solid Organ Transplant Recipients

eTable 5. Risk Factors for Influenza Occurrence in Solid Organ Transplant Recipients

eTable 6. Risk Factors for VZV Occurrence in Solid Organ Transplant Recipients

eTable 7. Association of VPI Occurrence and Graft-Loss/Death During the First 90 Days Post-Transplant

eFigure. Vaccine Preventable Infections in 4967 Solid Organ Transplant Recipients

This supplemental material has been provided by the authors to give readers additional information about their work.

© 2023 Walti LN et al. *JAMA Network Open.*

Abbreviations:

DNA, Deoxyribonucleic Acid

HBV, Hepatitis-B Virus

FOPH; Federal office of public health

NAAT, nucleic acid amplification tests

NGS, next-generation sequencing

PCR, polymerase chain reaction

STCS; Swiss Transplant Cohort Study

VPI, vaccine-preventable infection

eTable 1

**eTable1**	**Data sources (STCS/FOPH):**
In	The STCS prospectively enrolls all SOT recipients undergoing transplantation in Switzerland (Koller et al. Eur J Epidemiol (2013) 28:347–355, Stampf et al BMJ Open. 2021 Dec 15;11(12):e051176, Eur J Epidemiol (2013) 28:347–355). Designed as a patient-case system, the database captures both patient- and graft-specific data. Patients are followed from solid organ transplant (time zero) to graft loss and/or death whatever occurs first, and censored at last available follow up. Clinical and laboratory data are continuously collected and entered in the database at the time of transplantation, at 6 and 12 months, then yearly thereafter. Date of event was date of laboratory confirmation. Local site data managers are responsible for data collection pertaining to a certain follow-up period (e.g. baseline, 6 month, yearly). All infections were identified by transplant–infectious disease physicians using electronic hospital records and referral documentation for both inpatients and outpatients in intervals ranging from twice a week to every 3 months, according to standardized definitions (van Delden et al. Clin Infect Dis. 2020 Oct 1; 71(7): e159–e169.).
Federal Office of Public Health (FOPH):	In Switzerland, all healthcare workers and laboratories, operate according to the "diagnosing party" responsible for reporting' principle. Infectious diseases with reporting obligations are directly reported to the respective health authority using a declaration form for communicable diseases: (https://www.bag.admin.ch/bag/en/home/krankheiten/infektionskrankheiten-bekaempfen/meldesysteme-infektionskrankheiten/meldepflichtige-ik.html

eTable 2:

© 2023 Walti LN et al. *JAMA Network Open.*

eTable 2: VPI definitions		
	**Swiss Transplant Cohort Study**	**Swiss Federal Office of Public Health**
Hepatitis A	Detection of antibodies (IgM/IgG) or virus replication (PCR, culture, antigen) in a previously seronegative patient.	At least one of the following positive:<br>I. NAAT (PCR, Sequencing, NGS) Serum or Stool or<br>II. Stool Antigen or III. Antibody detection (IgM, Titer increase ≥ 4x or Seroconversion)
Hepatitis B	Documented post-transplantation new positive HBV-DNA by PCR or HbsAg in blood or compatible histology compatible with HBV infectious disease. Patient should not be known for chronic hepatitis B, inactive HbsAg or resolved hepatitis B	At least one of the following positive<br>I. NAAT (PCR, Sequencing, NGS) or<br>II. Antibody detection (anti-HBc-IgM) or<br>III. Antigen detection (AgHBs, AgHBe)
Diphtheria	Pathogen isolated + clinical signs and/or symptoms +<br>treatment given.	Isolation of Corynebacterium diphtheriae and other toxin-forming Corynebacteria (C. ulcerans, C. pseudotuberculosis)
Invasive *Haemophilus influenzae*	Pathogen isolated form a sterile site + clinical signs and/or symptoms +<br>treatment given.	At least one of the following positive (samples from sterile sites only):<br>I. Culture or<br>II. NAAT (PCR, Sequencing, NGS)
Influenza	Positive PCR/culture from a respiratory sample.	At least one of the following positive:<br>I. Culture or<br>II. NAAT (PCR, Sequencing, NGS) or<br>III. Antibody detection or<br>IV. Antigen detection<br>Not positive rapid antigen test.
Measles	Detection of antibodies (IgM/IgG) or virus replication (PCR, culture) in a previously seronegative patient.	At least of the following positive<br>I. Culture or<br>II. NAAT (PCR, Sequencing, NGS) or<br>III. Antibodies (IgM, ≥ 4 x titer increase<br>or Seroconversion).
Mumps	Detection of antibodies (IgM/IgG) or virus replication (PCR, culture, antigen, in situ hybridization) in a previously seronegative patient.	NA
Pertussis	Pathogen isolated + clinical signs and/or symptoms +<br>treatment given.	NA

© 2023 Walti LN et al. *JAMA Network Open.*

Invasive pneumococcal disease	Pathogen isolated from a sterile site + clinical signs and/or symptoms + treatment given.	At least one of the following positive (samples from sterile sites only):<br>I. Culture<br>II. NAAT (PCR, Sequencing, NGS) Serum or Stool or<br>III. Antibody detection (IgM, Titer increase ≥ 4x or Seroconversion)<br>IV. Antigen detection
Poliomyelitis	Detection of antibodies (IgM/IgG) or virus replication (PCR, culture, antigen) in a previously seronegative patient.	At least one of the following positive:<br>I. Culture<br>II. NAAT (PCR, Sequencing, NGS)<br>III. Antibody detection (relevant titer increase ≥ 4 x or Seroconversion)
Invasive Meningococcal disease	Pathogen isolated form a sterile site + clinical signs and/or symptoms + treatment given.	At least one of the following positive (samples from sterile sites only):<br>I. Culture<br>II. NAAT (PCR, Sequencing, NGS) Serum or Stool or<br>III. Microscopy<br>IV. Antigen detection
Rubella	Detection of antibodies (IgM/IgG) or virus replication (PCR, culture, antigen) in a previously seronegative patient.	At least one of the following positive:<br>I. Culture<br>II. NAAT (PCR, Sequencing, NGS)<br>III. Antibody detection (IgM, relevant titer increase or Seroconversion)
Tetanus	Pathogen isolated + clinical signs and/or symptoms +<br>treatment given.	At least one of the following positive:<br>I. Culture<br>II. NAAT (PCR, Sequencing, NGS)<br>III. Antibody detection (relevant titer increase ≥ 4 x or Seroconversion)
Tick-borne encephalitis	Detection of antibodies (IgM/IgG) or virus replication (PCR, culture, antigen) in a previously seronegative patient.	At least of the following positive<br>I. Culture or<br>II. NAAT (PCR, Sequencing, NGS) or<br>III. Antibodies (IgM, Titer increase ≥ 4 x<br>or Seroconversion).
Varicella zoster virus	Detection of virus replication and symptoms/signs of concurrent organ dysfunction.<br>Mucocutaneous infections: viral detection on vesicle swab (immunofluorescence, PCR, culture)	NA

© 2023 Walti LN et al. *JAMA Network Open.*

eTable 3

**eTable 3: Time from transplant to VPI occurrence**	
VPI	Median (IQR), months
Influenza	23.4 (6.9-47.9)
VZV	16.9 (6.2-41.7)
Hepatitis B	13.7 (13.1-32.7)
TBE	16.3 (NA)
IPD	19.0 (15.7-24.5)
IHI	7.76 (0.3-31.2)
Pertussis	44.8 (NA)
IMD	30.6 (NA)

Abbreviations: IMD, invasive meningococcal disease; IHI, invasive *Haemophilus influenzae* infection; IPD, invasive pneumococcal disease; IQR; interquartile range; NA, not applicable; TBE, tick-borne encephalitis; VPI, vaccine-preventable infection; VZV, varicella zoster virus.

eTable 4

**eTable 4 : Risk factors for VPI occurrence in solid organ transplant recipients**		
Variable	HR (95% CI)	p-value
Sex		
Male	Reference	
Female	1.15 (0.98-1.36)	0.08
Age, years		
<18	1.30 (0.94-1.79)	0.11
≥18-64.9	Reference	
≥65	1.22 (0.97-1.54)	0.09
Organ transplant		
Kidney	Reference	
Liver	0.59 (0.46-0.76)	<0.001
Lung	1.67 (1.31-2.15)	<0.001
Heart	1.35 (1.01-1.80)	0.04
Combined	0.73 (0.46-1.15)	0.17
Induction Therapy		
Basiliximab/Other	Reference	
ATG	1.06 (0.85-1.32)	0.60
Rituximab	1.17 (0.82-1.68)	0.39
Treated Rejection Episode		
No	Reference	
Yes	1.26 (0.90-1.76)	0.17

Abbreviations: ATG, antithymocyte globulins; CI, confidence interval; HR, hazard ratio; VPI, vaccine preventable infection.

eTable 5

**eTable 5 : Risk factors for influenza occurrence in solid organ transplant recipients**		
Variable	IRR (95% CI)	p-value

© 2023 Walti LN et al. *JAMA Network Open.*

Sex		
Male	Reference	
Female	0.971 (0.78-1.21)	0.79
Age, years		
<18	1.53 (0.97-2.41)	0.063
≥18-64.9	Reference	
≥65	1.25 (0.91-1.71)	0.09
Organ transplant		
Kidney	Reference	
Liver	0.53 (0.36-0.76)	0.001
Lung	2.51 (1.88-3.34)	<0.001
Heart	1.04 (0.66-1.63)	0.86
Combined	0.57 (0.31-1.09)	0.093
Induction Therapy		
Basiliximab/Other	Reference	
ATG	1.15 (0.85-1.58)	0.35
Rituximab	0.82 (0.47-1.42)	0.48

Abbreviations: ATG, antithymocyte globulins; CI, confidence interval; HR, hazard ratio.

eTable 6

eTable 6 : Risk factors for VZV occurrence in solid organ transplant recipients

Variable	IRR (95% CI)	p-value
Sex		
Male	Reference	
Female	1.32 (1.04-1.69)	0.024
Age, years		
<18	0.79 (0.47-1.35)	0.39
≥18-64.9	Reference	
≥65	1.19 (0.85-1.67)	0.30
Organ transplant		
Kidney	Reference	
Liver	0.59 (0.46-0.76)	0.006
Lung	0.71 (0.41-1.21)	0.21
Heart	1.72 (1.16-2.55)	0.007
Combined	0.77 (0.42-1.42)	0.39
Induction Therapy		
Basiliximab/Other	Reference	
ATG	0.92 (0.67-1.32)	0.59
Rituximab	1.38 (0.83-2.30)	0.20

Abbreviations: ATG, antithymocyte globulins; CI, confidence interval; HR, hazard ratio; VZV, varicella zoster virus.

eTable 7

eTable 7: Association of VPI occurrence and graft-loss/death during the first 90 days post-transplant

Variable	HR (95% CI)	P-value
Sex		
Male	Reference	

© 2023 Walti LN et al. *JAMA Network Open.*

Female	0.80 (0.70-0.91)	0.001
Age, years		
<18	0.61 (0.43-0.88)	0.008
≥18-64.9	Reference	
≥65	2.23 (2.03-2.76)	<0.001
Organ transplant		
Kidney	Reference	
Liver	1.59 (1.35-1.87)	<0.001
Lung	3.28 (2.75-3.89)	<0.001
Heart	1.44 (1.10-1.88)	0.007
Combined	1.53 (1.14-2.05)	0.005
VPI occurrence		
No	Reference	
Yes	2.44 (1.50-3.99)	<0.001

Abbreviations: CI, confidence interval; HR, hazard ratio; VPI, vaccine preventable infections

eFigure Vaccine preventable infections in 4967 solid organ transplant recipients.

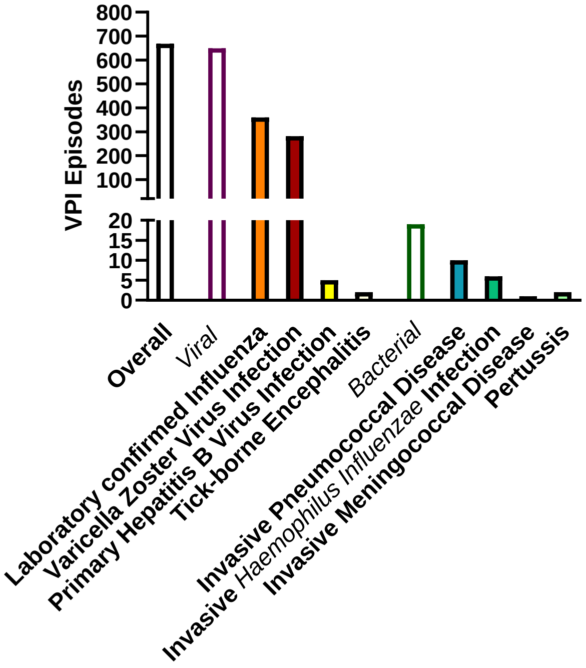

Cases of VPIs recorded in the STCS from May 2008 to December 2019. STCS; Swiss Transplant Cohort Study. VPI; vaccine preventable infection.

List of the members of the Swiss Transplant Cohort Study

Patrizia Amico, John-David Aubert, Vanessa Banz, Sonja Beckmann, Guido Beldi, Christoph Berger, Ekaterine Berishvili, Annalisa Berzigotti, Isabelle Binet, Pierre-Yves Bochud, Sanda Branca, Heiner Bucher, Emmanuelle Catana, Anne Cairoli, Yves Chalandon, Sabina De Geest, Olivier De Rougemont, Sophie De Seigneux, Michael Dickenmann, Joëlle Lynn Dreifuss, Michel Duchosal, Thomas Fehr, Sylvie

© 2023 Walti LN et al. *JAMA Network Open.*

Ferrari-Lacraz, Christian Garzoni, Déla Golshayan, Nicolas Goossens, Fadi Haidar, Jörg Halter, Dominik Heim, Christoph Hess, Sven Hillinger, Hans H Hirsch, Patricia Hirt, Günther Hofbauer, Uyen Huynh-Do, Franz Immer, Michael Koller, Mirjam Laager, Bettina Laesser, Frédéric Lamoth, Roger Lehmann, Alexander Leichtle, Oriol Manuel, Hans-Peter Marti, Michele Martinelli, Valérie McLin, Katell Mellac, Aurélia Merçay, Karin Mettler, Antonia Müller, Nicolas J Mueller, Ulrike Müller-Arndt, Beat Müllhaupt, Mirjam Nägeli, Graziano Oldani, Manuel Pascual, Jakob Passweg, Rosemarie Pazeller, Klara Posfay-Barbe, Juliane Rick, Anne Rosselet, Simona Rossi, Silvia Rothlin, Frank Ruschitzka, Thomas Schachtner, Urs Schanz, Stefan Schaub, Alexandra Scherrer, Aurelia Schnyder, Macé Schuurmans, Simon Schwab, Thierry Sengstag, Federico Simonetta, Susanne Stampf, Jürg Steiger, Guido Stirnimann, Ueli Stürzinger, Christian Van Delden, Jean-Pierre Venetz, Jean Villard, Julien Vionnet, Madeleine Wick, Markus Wilhelm, Patrick Yerly

© 2023 Walti LN et al. *JAMA Network Open.*
