# Supplementary material for: Vaccine-Preventable Infections Among Solid Organ Transplant Recipients in Switzerland
Source: JAMA Netw Open. 2023 Apr 28;6(4):e2310687. doi: 10.1001/jamanetworkopen.2023.10687 (PMC10148200; doi:10.1001/jamanetworkopen.2023.10687)
Supplement: Supplement 2. — Swiss Transplant Cohort Study [file jamanetwopen-e2310687-s002.pdf]

Supplemental Online Content: Nonauthor Collaborators
*First name, last name, and suffix (if applicable) are required and will appear in PubMed.

| *Group Name(s): Swiss Transplant Cohort Study | | | | | | | |
|---|---|---|---|---|---|---|---|
| ***First Name and Middle Initial(s)** | ***Last Name** | ***Suffix (eg, Jr, III)** | Academic Degrees | Institution | Location (city, state/province, country) | Role or Contribution, eg, chair, principal investigator | Group (if more than 1 Group listed in the byline) and/or Subgroup (eg, Steering Committee) |
| Patrizia | Amico | | | | | | |
| John-David | Aubert | | | | | | |
| Vanessa | Banz | | | | | | |
| Sonja | Beckmann | | | | | | |
| Guido | Beldi | | | | | | |
| Christoph | Berger | | | | | | |
| Ekaterine | Berishvili | | | | | | |
| Annalisa | Berzigotti | | | | | | |
| Isabelle | Binet | | | | | | |
| Pierre-Yves | Bochud | | | | | | |
| Sandra | Branca | | | | | | |
| Heiner | Bucher | | | | | | |
| Emanuelle | Catana | | | | | | |
| Anne | Cairoli | | | | | | |
| Yves | Chalandon | | | | | | |
| Sabina | De Geest | | | | | | |
| Olivier | De Rougemont | | | | | | |
| Sophie | De Seigneuex | | | | | | |
| Michael | Dickenmann | | | | | | |
| Joëlle Lynn | Dreifuss | | | | | | |
| Michel | Duchosal | | | | | | |
| Thomas | Fehr | | | | | | |
| Sylvie | Ferrari-Lacraz | | | | | | |
| Christian | Garzoni | | | | | | |
| Déla | Golshayan | | | | | | |
| Nicolas | Goossens | | | | | | |
| Fadi | Haidar | | | | | | |
| Jörg | Halter | | | | | | |

Supplemental Online Content: Nonauthor Collaborators
*First name, last name, and suffix (if applicable) are required and will appear in PubMed.

| ***First Name and Middle Initial(s)** | ***Last Name** | ***Suffix (eg, Jr, III)** | Academic Degrees | Institution | Location (city, state/province, country) | Role or Contribution, eg, chair, principal investigator | Group (if more than 1 Group listed in the byline) and/or Subgroup (eg, Steering Committee) |
|---|---|---|---|---|---|---|---|
| Dominik | Heim | | | | | | |
| Christoph | Hess | | | | | | |
| Sven | Hillinger | | | | | | |
| Hans H. | Hirsch | | | | | | |
| Patricia | Hirt | | | | | | |
| Günther | Hofbauer | | | | | | |
| Uyen | Huynh-Do | | | | | | |
| Franz | Immer | | | | | | |
| Michael | Koller | | | | | | |
| Mirjam | Laager | | | | | | |
| Bettina | Laesser | | | | | | |
| Frédéric | Lamoth | | | | | | |
| Roger | Lehmann | | | | | | |
| Alexander | Leichtle | | | | | | |
| Oriol | Manuel | | | | | | |
| Hans-Peter | Marti | | | | | | |
| Michele | Martinelli | | | | | | |
| Valérie | McLin | | | | | | |
| Katell | Mellac | | | | | | |
| Aurélia | Mercay | | | | | | |
| Karin | Mettler | | | | | | |
| Antonia | Müller | | | | | | |
| Nicolas J. | Müller | | | | | | |
| Ulrike | Müller-Arndt | | | | | | |
| Beat | Müllhaupt | | | | | | |
| Mirjam | Nägeli | | | | | | |
| Graziano | Oldani | | | | | | |
| Manuel | Pascual | | | | | | |
| Jakob | Passweg | | | | | | |
| Rosemarie | Pazeller | | | | | | |
| Klara | Posfay-Barbe | | | | | | |

Supplemental Online Content: Nonauthor Collaborators
*First name, last name, and suffix (if applicable) are required and will appear in PubMed.

| ***First Name and Middle Initial(s)** | ***Last Name** | ***Suffix (eg, Jr, III)** | Academic Degrees | Institution | Location (city, state/province, country) | Role or Contribution, eg, chair, principal investigator | Group (if more than 1 Group listed in the byline) and/or Subgroup (eg, Steering Committee) |
|---|---|---|---|---|---|---|---|
| Juliane | Rick | | | | | | |
| Anne | Rosselet | | | | | | |
| Simona | Rossi | | | | | | |
| Silvia | Rothlin | | | | | | |
| Frank | Ruschitzka | | | | | | |
| Thomas | Schachtner | | | | | | |
| Urs | Schranz | | | | | | |
| Stefan | Schaub | | | | | | |
| Alexandra | Scherrer | | | | | | |
| Aurelia | Schnyder | | | | | | |
| Macé | Schuurmans | | | | | | |
| Simon | Schwab | | | | | | |
| Thierry | Sengstag | | | | | | |
| Federico | Simonetta | | | | | | |
| Susanne | Stampf | | | | | | |
| Jürg | Steiger | | | | | | |
| Guido | Stirnimann | | | | | | |
| Ueli | Stützinger | | | | | | |
| Christian | Van Delden | | | | | | |
| Jean-Pierre | Venetz | | | | | | |
| Jean | Villard | | | | | | |
| Julien | Vionnet | | | | | | |
| Madelaine | Wick | | | | | | |
| Markus | Wilhelm | | | | | | |
| Patrick | Yerly | | | | | | |
