# Supplementary material for: Vaccine-Preventable Infections Among Solid Organ Transplant Recipients in Switzerland
Source: JAMA Netw Open. 2023 Apr 28;6(4):e2310687. doi: 10.1001/jamanetworkopen.2023.10687 (PMC10148200; doi:10.1001/jamanetworkopen.2023.10687)
Supplement: Supplement 3. — Data Sharing Statement [file jamanetwopen-e2310687-s003.pdf]

Data Sharing Statement

Walti. Vaccine-Preventable Infections Among Solid Organ Transplant Recipients in Switzerland. *JAMA Netw Open*. Published April 28, 2023.
doi:10.1001/jamanetworkopen.2023.10687

Data
Data available: No

Additional Information
Explanation for why data not available: Data will be shared upon reasonable request to the corresponding author.
